# The role of N-linked glycosylation in proteolytic processing and cell surface transport of the Cedar virus fusion protein

**DOI:** 10.1186/s12985-022-01864-5

**Published:** 2022-08-23

**Authors:** Kerstin Fischer, Selin Topallar, Franziska Kraatz, Martin H. Groschup, Sandra Diederich

**Affiliations:** Institute of Novel and Emerging Infectious Diseases, Friedrich-Loeffler-Institut, Federal Research Institute for Animal Health, Südufer 10, 17493 Greifswald-Insel Riems, Germany

**Keywords:** Cedar virus, Fusion protein, N-glycosylation, Biological activity

## Abstract

**Background:**

N-linked glycans on viral glycoproteins have been shown to be important for protein expression, processing and intracellular transport. The fusion glycoprotein F of Cedar virus (CedV) contains six potential N-glycosylation sites.

**Findings:**

To investigate their impact on cell surface transport, proteolytic cleavage and biological activity, we disrupted the consensus sequences by conservative mutations (Asn to Gln) and found that five of the six potential N-glycosylation sites are actually utilized. The individual removal of N-glycan g1 (N66), g2 (N79) and g3 (N98) in the CedV F_2_ subunit had no or only little effect on cell surface transport, proteolytic cleavage and fusion activity of CedV F. Interestingly, removal of N-linked glycan g6 (N463) in the F_1_ subunit resulted in reduced cell surface expression but slightly increased fusogenicity upon co-expression with the CedV receptor-binding protein G. Most prominent effects however were observed for the disruption of N-glycosylation motif g4 (N413), which significantly impaired the transport of CedV F to the cell surface, thereby also affecting proteolytic cleavage and fusion activity.

**Conclusions:**

Our findings indicate that the individual N-linked modifications, with the exception of glycan g4, are dispensable for processing of CedV F protein in transfection experiments. However, removal of g4 led to a phenotype that was strongly impaired concerning cell surface expression and proteolytic activation.

**Supplementary Information:**

The online version contains supplementary material available at 10.1186/s12985-022-01864-5.

## Introduction

Cedar virus (CedV) is a henipavirus within the *Paramyxoviridae* family that was first isolated from an Australian Pteropus colony in 2012 [1]. While other members of this genus like Hendra (HeV) and Nipah virus (NiV) are classified as highly pathogenic BSL4 agents, experimental studies of CedV in small animal models have only resulted in asymptomatic infections so far [1, 2]. Thus, recent research has focused on deciphering viral and cellular factors that may contribute to the differences in pathogenicity of these closely related viruses.

The two structural glycoproteins in the henipavirus envelope, the fusion protein F and the attachment protein G play an essential role in the overall pathogenesis, e.g. by determining the tissue tropism of the virus. After receptor binding by the receptor-binding protein G, both proteins act in concert to mediate pH-independent fusion of the viral and cellular membrane so that the virus can release the genome into the host cell [3–5]. Later during the replication cycle, their interaction enables cell-to-cell fusion of receptor-bearing neighboring cells resulting in the formation of multinucleated giant cells, so called syncytia, which are fundamental for virus spread in tissues, and thus greatly contribute to pathogenesis.

A prerequisite for fusion through glycoprotein interactions is the presence of properly folded and processed viral glycoproteins at the cell surface including proteolytically activated F proteins [3, 4]. Since viruses employ the host cell machinery to process their proteins, the conformational integrity of the viral envelope glycoproteins may be affected by N-linked glycosylation, one of the most common types of post-translational membrane protein modifications that enzymatically links oligosaccharides to certain asparagine (N) residues at N-glycosylation consensus sites (N-X-S/T, in which X can be any amino acid except proline) in the endoplasmic reticulum [6]. However, even though the N-X-S/T motif is a prerequisite for N-glycosylation, it is not sufficient, as not all ectodomain N-glycosylation consensus sites are N-glycosylated. A stable conformation adoption, localization of the protein and N-X-S/T site accessibility to solvent are described to play a role in deciding whether a site is N-glycosylated or not [7–9]. N-glycosylation has been shown to influence correct protein folding, processing as well as intracellular and surface transport of several viral envelope glycoproteins such as the influenza virus hemagglutinin [10, 11], pseudorabies virus glycoprotein gH [12] and the Lassa virus glycoprotein GP-C [13]. Consequently, N-glycans can have a significant impact on the biological function of the envelope protein affecting viral particle assembly, receptor binding and eventually, virus entry into host cells [14–17]. Moreover, N-glycans may influence the antigenicity of a virus by physically shielding antigenic sites, thus preventing antibody recognition and subsequent antibody-mediated neutralization [16, 18, 19].

A number of fusogenic viral glycoproteins have been described to rely on N-glycosylation for proteolytic processing and, consequently, fusion activity [13, 15, 20]. Interestingly, N-glycans on the Ebola virus glycoprotein GP do not seem to play an important role for cell surface expression of GP but rather affect protease sensitivity and thus processing and biological activity of the protein [20]. In line with the function of N-glycans for other viral glycoproteins, N-glycans on paramyxovirus F glycoproteins have an impact on protein expression, processing and transport to the cell surface but also influence fusogenicity and production of infectious virus progeny [9, 21–23]. Previous studies on NiV and HeV F revealed that both proteins are heavily glycosylated but appeared to be comparably resistant to the effects of single N-glycan removal [24–26].

Cedar virus fusion protein F contains six potential N-glycosylation sites. In this study, we determined the glycosylation site usage and analyzed the N-glycans for their functional relevance. We provide evidence that five of six N-glycosylation sites undergo N-glycan attachment. Besides one N-glycan mutant that failed to reach the cell surface and therefore remained uncleaved and fusion-defective, individual removal of the other N-glycans had no or only little effect on F protein expression, processing and biological activity.

## Methods

### Cell lines and transfection

Vero76 and MDCK-2 cells (Collection of Cell Lines in Veterinary Medicine, Friedrich-Loeffler-Institut, FLI; CCLV-RIE 0228 and 1061) were maintained in Dulbecco’s modified Eagle medium (DMEM) supplemented with 10% fetal calf serum (FCS) and incubated at 37 °C and 5% CO_2_. Cells were reverse transfected in Opti-MEM (Gibco) using Lipfoctamine 3000 (Invitrogen) according to the manufacturer’s instructions.

### Plasmids and site-directed mutagenesis

The open reading frames (ORF) of CedV F and G (GenBank accession no. NC_025351.1) were synthesized by GeneArt (Thermo Fisher Scientific Inc.) and subcloned into the pCAGGS expression vector (kindly provided by Stefan Finke, Friedrich-Loeffler-Institut; [27]). The ORF of the CedV F gene was codon-optimized for expression in human cells and HA-tagged at the C-terminus (see Additional file 1 for sequence information). CedV F N-glycosylation mutants g1-g6 as well as mutants F g4AST and F g4NSA (see Table 1) were generated by site-directed mutagenesis using either the QuikChange Lightning Site-Directed Mutagenesis Kit (Agilent) or the Phusion High-Fidelity DNA polymerase (ThermoScientific). Sequences of all constructs were confirmed by SANGER sequencing. Primers were designed according to manufacturer’s instructions. Primer sequences are available on request.

**Table 1:** CedV F N-glycosylation mutants

	g1	g2	g3	g4	g6	g5
	66 67 68	79 80 81	98 99 100	413 414 415	463 464 465	484 485 486
F	N I T	N E T	N N T	N S T	N Q S	N I S
g1	**Q** I T	N E T	N N T	N S T	N Q S	N I S
g2	N I T	**Q** E T	N N T	N S T	N Q S	N I S
g3	N I T	N E T	**Q** N T	N S T	N Q S	N I S
g4	N I T	N E T	N N T	**Q** S T	N Q S	N I S
g6	N I T	N E T	N N T	N S T	**Q** Q S	N I S
g5	N I T	N E T	N N T	N S T	N Q S	**Q** I S
g4_AST_	N I T	N E T	N N T	**A** S T	N Q S	N I S
g4_NSA_	N I T	N E T	N N T	N S **A**	Q S N	N I S

Numbers indicate amino acid position. Boldfaced, underlined characters highlight the generated mutations

### Colocalization studies with DS Red2-ER

2 × 10^5^ MDCK-2 cells/24-well were co-transfected with plasmids coding for CedV F or F mutants and the plasmid pDS Red2-ER (Clontech Laboratories; kindly provided by Dr. Birke Tews, Friedrich-Loeffler-Institut). At 24 h p.t., cells were fixed with 2% paraformaldehyde and permeabilized with 0.2% Triton-X 100/PBS. Then, cells were incubated with a polyclonal anti-HA tag antibody (H6908, Sigma; 1:500 dilution in 0.35% BSA/PBS) for 1 h at 4 °C. After washing, a goat anti-rabbit Alexa Fluor 488 (1:500; LifeTechnologies) was added for 45 min at 4 °C. Cell nuclei were counterstained with 4′,6-Diamidin-2-phenylindol (DAPI). Representative images were recorded with a confocal laser scanning microscope (Leica SP5) and processed with the ImageJ software version 1.45 s [28].

### Metabolic labeling and immunoprecipitation

For pulse-chase analysis, 1 × 10^6^ MDCK-2 cells per 35 mm dish were reverse transfected with plasmids encoding CedV F or mutant CedV F proteins. At 24 h p.t., metabolic labeling and immunoprecipitation were performed as described previously [29]. Precipitated proteins were separated on a 12% polyacrylamide gel under reducing conditions. Dried gels were subjected to autoradiography and analyzed with a CR35 Dark Box Image analyser (Duerr Medical) and AIDA Imager Analyser 5. For treatment with glycosidases, samples were suspended in sample buffer (0.5 M Tris/HCl pH 6.8, SDS, glycerin, bromophenol blue, dH_2_O) and then treated with N-glycosidase F (PNGase F; NEB) according to the instructions of the manufacturer, or left untreated (no enzyme added). Then, samples were analyzed by SDS-PAGE and autoradiography as described above.

### Surface biotinylation and Western blot analysis

In order to analyze cell surface expression of CedV F or mutant F proteins, cell surface biotinylation with subsequent Western blot analyses were performed as described elsewhere [29].

For treatment with glycosidases, samples were suspended in sample buffer and then treated with endo-β-N-acetylglucosaminidase H (Endo H; NEB) according to the instructions of the manufacturer, or left untreated (no enzyme added). Then, samples were analyzed by SDS-PAGE and Western blot analysis.

### Fusion assay

To analyze biological activity of F and mutant F proteins, a total of 3 × 10^5^ Vero76 cells or 2 × 10^5^ MDCK-2 cells per 24-well were co-transfected with expression plasmids encoding CedV G and either CedV F or mutant F proteins at the ratio of 3:1, respectively. At 30 h p.t., cells were fixed with ethanol and then stained with 1:10 Giemsa solution. Representative images were recorded using a Nikon Eclipse TS100 with IC-Capture (200 × magnification).

### Luciferase reporter gene-based quantitative fusion assay

To quantify fusion activity of CedV F or mutant F proteins upon co-expression with CedV G protein, a renilla luciferase-gene based quantitative fusion assay was performed as described previously [29]. Briefly, Vero76 cells were co-transfected with plasmids encoding for the indicated CedV glycoproteins F and G as well as a pCITE Renilla plasmid containing the luciferase gene under control of a T7 promotor (pCite2a obtained from Novagen) or with pCAGGS T7 plasmid (kindly provided by Thomas Hoenen, Friedrich-Loeffler-Institut; [30]). At 24 h p.t., Vero76 cells expressing the T7 polymerase were layered on the glycoprotein-expressing cells and incubated for 3 h at 37 °C. Then, cells were lysed and luciferase activity measured using a luminometer. Reporter activity measured for the parental CedV F protein co-transfected with CedV G protein was set to 1 serving as a reference point for fusion activity. Background activity of the luciferase reporter was assessed with cells transfected with pCAGGS CedV G and pCITE Renilla only, layered with T7 polymerase expressing cells.

### Statistical analysis

Statistical analyses were performed using GraphPad Prism version 9.0.0. The parametric Student’s t-test was used to evaluate the data. Statistical significance is represented as: (*) = *p* ≤ 0.05, (**) = *p* ≤ 0.005.

## Results

### Five of six potential N-linked glycosylation sites in the CedV F protein are N-glycosylated

**Fig. 1:**
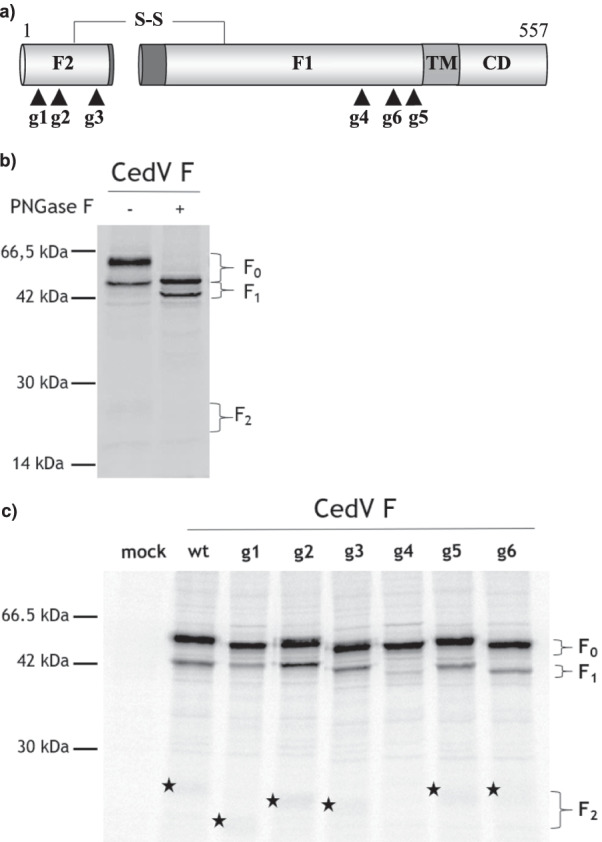
Analysis of N-linked glycosylation in CedV F protein. **a** Schematic of localization of potential N-glycans. N-glycosylation mutants are named g1–g6. Black triangles indicate potential N-glycosylation sites within the F1 and F2 subunits. TM: transmembrane domain; CD: cytoplasmic domain; **b** PNGase F digest of CedV F protein. MDCK cells expressing CedV F protein were metabolically labeled for 15 min (pulse) and then incubated for 2 h in serum-free nonradioactive medium (chase). After immunoprecipitation of F proteins from cell lysates, samples were suspended in sample buffer and then treated with N-glycosidase F (PNGase F) according to the instructions of the manufacturer, or left untreated. After separation on a 12% SDS-gel under reducing conditions, samples were analyzed by autoradiography; **c** Proteolytic processing of CedV F and F mutants. MDCK cells expressing F proteins are metabolically labeled as described above. After immunoprecipitation of F proteins from cell lysates and separation on a 12% SDS-gel under reducing conditions, samples were analyzed by autoradiography. Molecular masses of marker proteins are indicated. n = 2. Stars indicate the F_2_ subunit

CedV F protein contains six potential N-linked glycosylation sites, three sites (g1, g2, g3) in the F_2_ subunit and three (g4, g5, g6) in the F_1_ subunit (Fig. 1a). Initially, to examine if the CedV F protein contains N-glycan modifications, we combined a pulse-chase analysis with a PNGase F digest, which removes all N-linked glycans. Therefore, radiolabeled cell lysates from MDCK cells expressing parental CedV F (in the following indicated as wild-type wt) were immunoprecipitated and subsequently incubated with PNGase F or left untreated. Samples were then analyzed in a 12% SDS gel to visualize the relative mobility of the precursor F_0_ and the subunits F_1_ and F_2_. A significant shift in mobility of the F_0_ precursor as well as of the F_1_ subunit was observed after PNGase F treatment (Fig. 1b) indicating that CedV F protein is indeed glycosylated.

In order to further investigate the individual N-glycan sites, we next introduced single conservative asparagine-to-glutamine changes (N to Q) in position 1 of the N-glycan recognition sequence NxS/T using site-directed mutagenesis (Fig. 1a). We then analyzed expression and mobility of the wt and mutant CedV F proteins in another pulse-chase experiment. Again, radiolabeled cell lysates from MDCK cells expressing either wt CedV F or g1-g6 mutants were immunoprecipitated and analyzed on a 12% SDS gel. Analysis of the precursor F_0_ and the subunits F_1_ and F_2_ protein in Fig. 1c revealed that all CedV F N-glycan mutants were expressed at levels similar to the wt CedV F protein. Compared to the parental CedV F, no change in mobility was seen for the F_1_ subunit of the g5 mutant, which strongly suggests that the g5 site is not N-glycosylated. In contrast, slight mobility shifts exhibited by the F_0_ precursors of the other five mutants (g1, g2, g3, g4, g6) point towards N-glycan modifications at the respective sites. In line with the faster mobility of the mutant F_0_ precursors, the F_1_ subunit of mutant g6 and the F_2_ subunit of mutants g1, g2 and g3 migrated faster than the wt CedV F protein subunits (Fig. 1c). The differences in mobility between the F_2_ subunits of g1, g2 and g3 are likely due to variation in N-linked carbohydrates. The F_1_ subunit of the g3 appears to be slightly shifted, however, this is due to the performance of the gel and a shift of g3 F1 was ruled out in repeats of the experiment. For mutant g4, a slight mobility shift of F_0_ was observed indicating that changes to the N-glycosylation site N413 are consistent with a lack of glycosylation. Interestingly, no proteolytic cleavage into mature F_1_ and F_2_ subunits was detectable for this g4 mutant leading to the assumption that changes to the g4 N-glycosylation site result in impaired proteolytic processing. In contrast, all other mutants were processed as efficiently as wt CedV F (Fig. 1c).

### N-glycosylation at N413 (g4) affects intracellular trafficking and proteolytic processing of CedV F protein

**Fig. 2:**
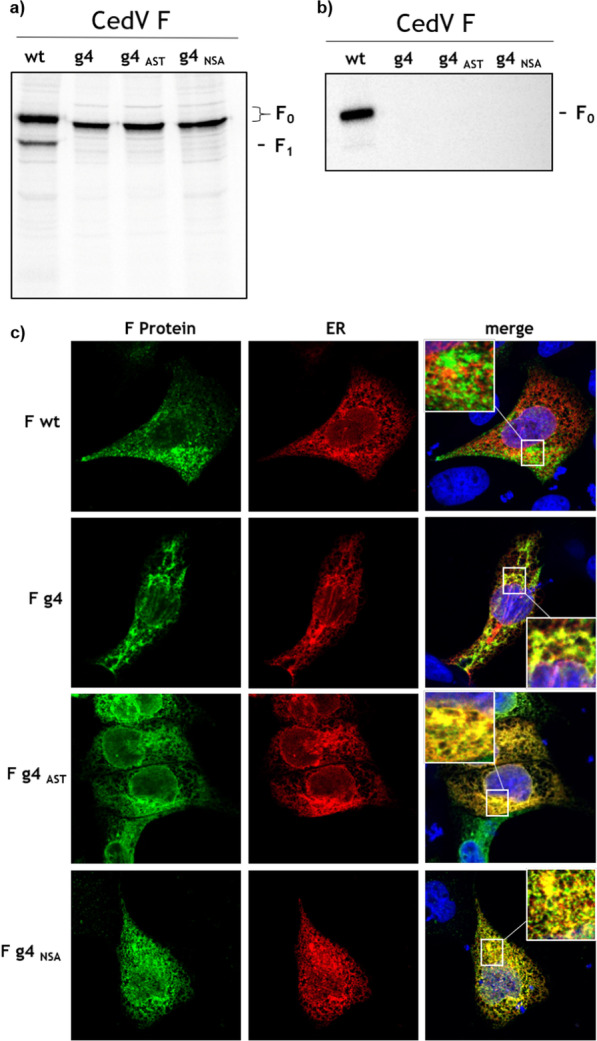
Analysis of amino acid substitutions at N-glycan consensus sequence 413–415 (g4) for expression. **a** Cells were transfected with either the wt F, g4, g4AST or the g4NSA gene. At 24 h p.t., cells were metabolically labeled for 15 min (pulse) and then incubated for 2 h in serum-free nonradioactive medium (chase). After immunoprecipitation of F proteins from cell lysates and separation on a 12% SDS-gel under reducing conditions, samples were analyzed by autoradiography. wt: wild-type; **b** Cell surface expression of CedV F proteins. Cells were transfected with either wt F, g4, g4AST or the g4NSA gene. At 24 h p.t., MDCK-2 cells expressing F proteins were surface-labeled with biotin on ice. After cell lysis, biotinylated proteins were immunoprecipitated using NeutrAvidin beads and subjected to SDS-PAGE under non-reducing conditions. Precipitated F proteins were visualized using an antibody against the HA-tag (H6908), HRP-labeled secondary antibodies and chemiluminescence. Representative blots are shown from four independent experiments. **c** Intracellular localization of wt and mutant CedV F proteins in MDCK-2 cells. F proteins are stained with anti-HA-tag specific primary antibodies and AlexaFluor488-conjugated secondary antibodies. The endoplasmic reticulum (ER) was visualized using a pDS Red2-ER plasmid-derived red fluorescent labeling. Representative images from two independent experiments are displayed. Inserts show magnifications of indicated areas. Magnification, × 63

We have previously shown that cell surface transport followed by endocytosis is critical for proteolytic cleavage of CedV F [29]. For mutant CedV F g4, we thus hypothesized that cell surface transport, and thus maturation, was critically impaired and that either the lack of N-glycosylation at the specific position or the particular asparagine-to-glutamine change would lead to intracellular retention. To test this, we generated two more mutants in which the asparagine (N413) or the threonine (T415) were replaced by an alanine residue (mutant g4AST and g4NSA, respectively) resulting in the loss of the N-glycan modification site. We first analyzed both mutants for their expression, migration and processing in a pulse chase experiment and found that they displayed a similar phenotype as g4. Accordingly, a slight mobility shift of the F_0_ protein but no proteolytic cleavage into F_1_ and F_2_ subunits was seen for the g4, g4AST and g4NSA mutants (Fig. 2a). Because cell surface expression of CedV F followed by endocytosis plays a critical role in proteolytic activation of henipavirus F proteins, we next investigated these cleavage-impaired g4 mutants for their cell surface expression in a surface biotinylation assay followed by Western blot analysis under non-reducing conditions [29, 31, 32]. Interestingly, in contrast to the wt F protein, none of the g4 mutants were detected at the cell surface (Fig. 2b). In agreement with these findings, a co-immunofluorescence analysis revealed that the g4 mutants largely co-localized with a red fluorescent cellular compartment marker for the endoplasmic reticulum (ER; Fig. 2c) indicating the accumulation of the mutants in the ER. Noteworthy, the wt F protein was barely detected to co-localize with the ER. Since cell surface expression of g4 could not be rescued by alanine substitutions within the N-glycan recognition sequence, our data point towards the importance of the actual N-glycan at this specific position to promote cell surface transport.

### Removal of individual N-glycans does not impair cell surface expression or proteolytic processing of CedV F protein

**Fig. 3:**
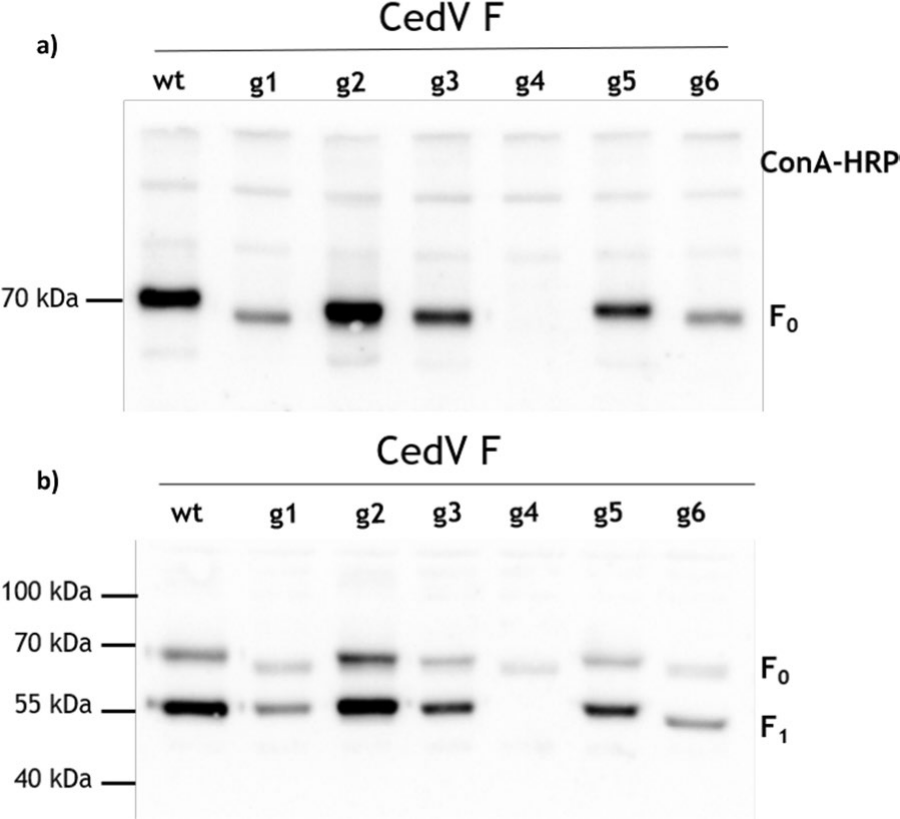
Cell surface expression of CedV F proteins. At 24 h p.t., MDCK-2 cells expressing F proteins were surface-labeled with biotin on ice. After cell lysis, biotinylated proteins were immunoprecipitated using NeutrAvidin beads and subjected to SDS-PAGE under **a** non-reducing and **b** reducing conditions (n = 2). Precipitated F proteins were visualized using an antibody against the HA-tag (H6908), HRP-labeled secondary antibodies and chemiluminescence. In **a**, ConA staining is used as a loading control. Molecular masses of marker proteins are indicated

Since N-glycosylation has been known to be critical for proper protein folding and intracellular trafficking, we next aimed to investigate the cell surface expression of the other N-glycosylation mutants. Therefore, we biotinylated the cell surface of MDCK cells expressing either wt CedV F or the N-glycan mutants and assessed the level of cell surface expression and processing by NeutrAvidin immunoprecipitation followed by Western blot analysis under non-reducing and reducing conditions (Fig. 3a and b, respectively). Apart from CedV F g4, all mutant F proteins were expressed at the cell surface (Fig. 3a) and showed to be proteolytically cleaved (Fig. 3b). ConA staining of biotinylated cell surface proteins served as a loading control (Fig. 3a) and revealed equal amounts of total protein loaded per lane. In agreement with our findings in the pulse-chase experiment, mobility shifts of the mutant F_0_ precursors were observed for all but one, further confirming that mutating the predicted N-glycosylation site of mutant CedV F g5 is not affecting N-glycosylation (Fig. 3a, b).

Interestingly, in comparison to the wt F, a marked decrease in the level of surface expression was noted for g1 and g6, while expression of g3 and g5 was only slightly reduced (Fig. 3a). In contrast, surface expression of g2 was enhanced in comparison to wt F protein. However, all CedV F_0_ precursor proteins, with the exception of g4 mutant as described above, were efficiently cleaved into the fusion-active F_1_/F_2_ subunits (Fig. 3b).

### Removal of certain N-glycans on CedV F results in moderately increased fusogenicity

**Fig. 4:**
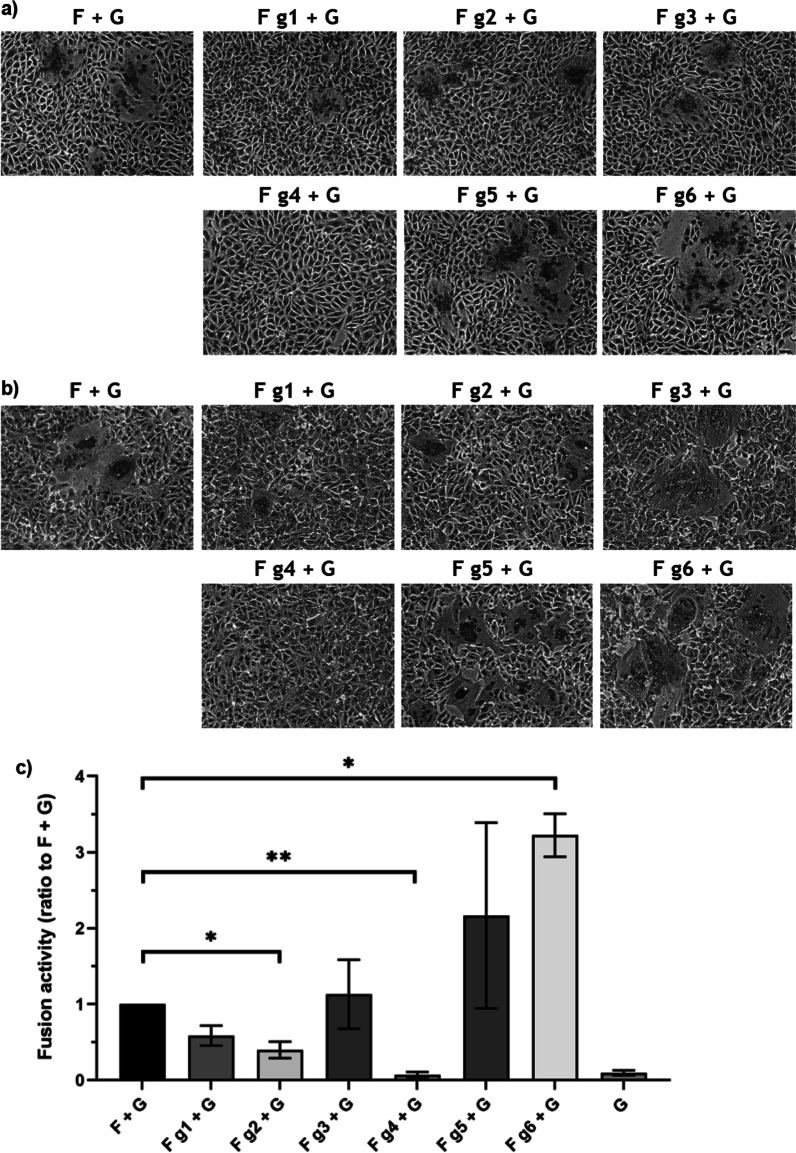
CedV F glycoprotein-mediated fusion activity. Syncytium formation in **a** MDCK-2 and **b** Vero76 cells co-expressing CedV F and G proteins was visualized by Giemsa staining at 30 h p.t.. Magnification, × 20. n = 3; **c** Quantitative reporter gene assay. At 24 h p.t., Vero76 cells expressing the T7 polymerase were layered on the cells expressing the indicated glycoproteins and incubated for 3 h at 37 °C. After cell lysis, luciferase activity was measured. Samples were tested in duplicates in three independent experiments. Reporter activity measured for the parental CedV F protein co-transfected with CedV G protein was set to 1 serving as a reference point for fusion activity. Bars represent the fusion activities of the different (mutant) CedV F proteins in relation to the fusion activity of the parental protein and include the standard error of the mean (SEM). Statistical analysis: unpaired Student’s *t*-test with Welch’s correction; (*) *p* ≤ 0.05; (**) *p* ≤ 0.005

Since removal/destruction of most N-glycosylation consensus motifs had little or no effect on F0 processing or cell surface expression, we next aimed to assess whether mutations consistent with a lack of particular N-glycosylation had any effects on the fusogenicity of CedV F. Therefore, we performed a standard fusion assay in permissive Vero and MDCK cells that were co-transfected with wt CedV G and CedV F wt or mutants for 30 h. Representative images of syncytium formation exhibited by wt CedV F and the g1 to g6 mutants are shown in Fig. 4a, b. As expected, the strongly impaired cell surface expression and lack of proteolytic activation of mutant g4 correlated with a marked decrease in fusogenicity in both cell lines. Reduced cell surface expression of CedV F g1 equally resulted in a decrease in fusion activity. However, mutant g2 with a rather wt-like cell surface expression or even slightly enhanced surface expression also displayed a slightly reduced fusion activity in comparison to the wt, indicating that cell-to-cell fusion is a multifactorial process and not only determined by cell surface availability of the F protein. In this context, and despite comparatively low cell surface expression of CedV F g6 (Fig. 3a, b), number and size of syncytia for mutant g6 seemed to be increased when compared to the wt CedV F protein (Fig. 4a, b). Quantification of fusion activity using a luciferase reporter-based assay in Vero76 cells however confirmed these differences. Reporter activity of mutant g6 was increased significantly (more than threefold, *p* value ≤ 0.05) in contrast to the wt CedV F protein, while only background level reporter activity was measured for fusion-deficient mutant g4 (*p* value ≤ 0.005; Fig. 4c). Also, fusion activity of F g2 was significantly reduced (*p* value ≤ 0.05) compared to wt F albeit the elevated surface expression of g2.

**Fig. 5:**
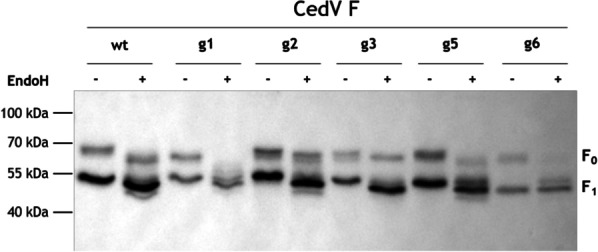
Analysis of N-linked carbohydrates in CedV F protein expressed at the cell surface of MDCK-2 cells. Surface biotinylated CedV F proteins were immunoprecipitated using NeutrAvidin beads and either left untreated (lanes “−”) or digested with endo-β-N-acetylglucosaminidase H (Endo H; lanes “+”). Samples were separated by SDS-PAGE under reducing conditions, and blots were analyzed using an antibody against the HA-tag (H6908), HRP-labeled secondary antibodies and chemiluminescence. Molecular masses of marker proteins are indicated. n = 2

Finally, to analyze the N-glycan composition of the CedV F protein at the cell surface, we combined a surface biotinylation assay with an Endo H digest, which results in the detachment of only high-mannose-type N-glycans. As shown in Fig. 5, endo H digestion resulted in a mobility shift of the wt CedV F_0_ precursor and the F_1_ subunit, suggesting the presence of at least one endo H-sensitive glycan. Since CedV F g6 was the only N-glycan mutant in our analysis without faster migration of its F_1_ subunit, we concluded that the missing glycan at position 463–465, which is present in all the other (mutant) CedV F proteins displaying a mobility shift, is endo H-sensitive.

In summary, our data indicate that five of six potential N-glycosylation sites within the CedV F protein are utilized. While four of these five N-glycans showed to be dispensable for CedV F processing, the glycan g4 was shown to be of importance for intracellular and cell surface transport, thus promoting further protein processing. Removal of the glycan g4 in the CedV F head domain resulted in the intracellular retention of this mutant and, subsequently, severe defects in fusion activity.

## Discussion

N-linked glycosylation of viral glycoproteins is known to play an important role in virus infection. Glycosylation can have a strong impact on protein processing, intracellular trafficking, cell surface transport, and conformational integrity of viral envelope proteins. Moreover, N-linked glycans may affect biological activity and immunogenicity, e.g. by shielding against neutralizing antibodies [9, 16, 26, 33, 34].

The F glycoproteins of HeV and NiV, two highly pathogenic henipaviruses, are known to be heavily glycosylated and specific N-glycans were demonstrated to be of functional importance during different steps of the viral life cycle [24, 25]. However, little is known about the role of N-glycosylation for the F glycoprotein of low pathogenic CedV. In this study, we thus aimed to investigate whether the CedV F protein is N-glycosylated and whether these N-glycans affect CedV F protein expression, processing and biological activity.

With respect to the number and occupancy of N-linked glycosylation sites and their functional relevance, there are striking similarities between the F proteins of the highly pathogenic NiV and HeV and the low pathogenic CedV. Overall, CedV F protein glycosylation mutants appeared to be relatively resistant to individual N-glycan removal. In line with our findings, most of the N-glycans in HeV and NiV F had rather limited functional effects on glycoprotein processing. However, one specific N-glycosylation site in the HeV F (N414) and NiV F protein (N414) in the F_1_ subunit was shown to affect expression and efficient cell surface transport [24–26]. Since henipavirus F glycoproteins are initially expressed as fusion-inactive precursors at the cell surface and require endocytosis for proteolytic activation [29, 31, 32], the impaired cell surface transport of the HeV and NiV g4 glycosylation mutants greatly impaired proteolytic processing and thus fusion activity [24, 25]. Similarly, the vast majority of fusion-deficient CedV F g4 was retained in the ER of transfected cells, as demonstrated by colocalization with an ER marker, which prevented further steps in processing including proteolytic cleavage.

CedV F and NiV F are both glycosylated at aa positions N413 and N414 [24], respectively. It is noteworthy that the corresponding amino acid sequence in HeV F generally does not undergo N-glycosylation, but is of similar importance for intracellular transport [25]. Moreover, analysis of NiV F protein also revealed that, besides the presence of the N-glycan, the actual aa sequence at position 414–416 plays a significant role for protein transport and processing [24]. While initial disruption of NiV F g4 with a conservative threonine to glycine change (T416G) resulted in the absence of the N-glycan and prevented cell surface transport, the substitution of asparagine with glutamine (N414Q) showed to rescue cell surface expression and cleavage of the mutant [24]. In contrast, all CedV F g4 mutants (g4, g4AST or g4NSA) generated in this study were retained intracellularly irrespective of the aa substitution. The introduced N-glycosylation site mutations N414A and T416A did not rescue cell surface transport, which prevented further steps in CedV F maturation. This indicates that, in contrast to highly pathogenic NiV and HeV, a functional N-glycosylation consensus site g4 (consistent with N-glycosylation) is critical for cell surface transport, and thus further processing and biological function of the protein.

The lack of certain N-glycans on surface glycoproteins of other paramyxoviruses like Newcastle disease virus, measles and Sendai virus resulted in severe fusion defects [21, 22, 35]. Glycan removal from the F_2_ subunit of CedV F only slightly decreased fusion activity (g1, g2) or did not affect it at all (g3). However, disruption of the CedV F N-glycosylation site g6 (N463Q), usually occupied with an N-glycan of the high mannose-type, resulted in a threefold increase in fusion activity in the quantitative fusion assay despite reduced cell surface expression of this mutant (Fig. 3a, b). This finding is consistent with the observed phenotype of the HeV F mutant N464A, which was considered hyperfusogenic [25]. The authors emphasized that this glycan is localized in the transmembrane-proximal heptad repeat region of HeV F_1_, for which glycosylation has also been shown to strongly impact membrane fusion activity of other paramyxoviruses such as Newcastle disease virus and human respiratory syncytial virus [21, 36].

As observed by Aguilar et al. (2006), glycosylation of individually expressed glycoproteins may vary between different cell lines depending on the glycosylation capacity of the individual cell type. Consequently, this variation may equally have an impact on fusogenicity of the glycan mutants in different cell lines as described for NiV F mutants in MDCK-2 and 293T cells [26]. The considerable variation in the glycosylation machinery among different cell lines is clearly a common limitation of many studies including this study. In addition, many analyses rely on data of overexpressed single proteins in transfection experiments. Thus, future confirmation of the observed effects in the viral context should be extended to study the role of N-glycosylation with regard to virus particle production, F-G interactions, and ultimately, virus infectivity. Moreover, future studies should investigate possible effects of N-glycans on immunogenicity. For example, the loss of certain N-glycans appeared to increase the susceptibility of NiV to antibody neutralization [26], suggesting a role in immune evasion by masking antigenic sites. Similar effects have been observed for glycoproteins of other viruses like HeV, HIV, Hepatitis C virus, Lassa virus and Influenza virus [14, 33, 37–40].

## Conclusion

In conclusion, our data indicate that five of six predicted N-linked glycosylation sites in CedV F are utilized in MDCK-2 cells (three in the F_2_ subunit: N66, N79, N98; and two in the F_1_ subunit: N413, N463) and that the N-linked glycan N413 is of particular importance for CedV F processing and transport to the cell surface. Mutation of the g4 N-glycosylation site had severe functional consequences, resulting in the lack of proteolytic cleavage and thus biological activity. While removal of other N-glycans alone had rather limited functional effects on glycoprotein processing and biological activity, it remains to be investigated if simultaneous removal of multiple N-glycans affects CedV F maturation and functionality more significantly.

## Supplementary Information

**Additional file 1:** Sequence information for codon-optimized CedV F HAtag gene. Start and stop codon are highlighted in bold and are underlined. CedV F gene was codon-optimized according to the human codon usage bias and synthesized by Gene Art. Additionally, the coding information for an HA-Tag was included. The CedV F gene was then subcloned into the pCAGGS expression vector using the restriction enzymes SacI and NheI (indicated in italics and underlined).

## Data Availability

The datasets used and/or analyzed in the current study are available from the corresponding author upon reasonable request.

## Abbreviations

NiV: Nipah virus
CedV: Cedar virus
BSL-4: Biosafety level-4
F protein: Fusion protein F
G protein: Glycoprotein G
wt: Wildtype
FCS: Fetal calf serum

## References

[1] Marsh GA, de Jong C, Barr JA, Tachedjian M, Smith C, Middleton D, Yu M, Todd S, Foord AJ, Haring V, Payne J, Robinson R, Broz I, Crameri G, Field HE, Wang LF (2012). Cedar virus: a novel henipavirus isolated from Australian bats. PLoS Pathog.

[2] Schountz T, Campbell C, Wagner K, Rovnak J, Martellaro C, DeBuysscher BL, Feldmann H, Prescott J (2019). Differential innate immune responses elicited by Nipah virus and cedar virus correlate with disparate in vivo pathogenesis in hamsters. Viruses.

[3] Chang A, Dutch RE (2012). Paramyxovirus fusion and entry: multiple paths to a common end. Viruses.

[4] Aguilar HC, Iorio RM (2012). Henipavirus membrane fusion and viral entry. Curr Top Microbiol Immunol.

[5] Navaratnarajah CK, Generous AR, Yousaf I, Cattaneo R (2020). Receptor-mediated cell entry of paramyxoviruses: mechanisms, and consequences for tropism and pathogenesis. J Biol Chem.

[6] Marshall RD (1972). Glycoproteins. Annu Rev Biochem.

[7] Bause E (1983). Structural requirements of N-glycosylation of proteins. Studies with proline peptides as conformational probes. Biochem J.

[8] Zielinska DF, Gnad F, Wisniewski JR, Mann M (2010). Precision mapping of an in vivo N-glycoproteome reveals rigid topological and sequence constraints. Cell.

[9] Ortega V, Stone JA, Contreras EM, Iorio RM, Aguilar HC (2019). Addicted to sugar: roles of glycans in the order Mononegavirales. Glycobiology.

[10] Gallagher PJ, Henneberry JM, Sambrook JF, Gething MJ (1992). Glycosylation requirements for intracellular transport and function of the hemagglutinin of influenza virus. J Virol.

[11] Klenk HD, Wagner R, Heuer D, Wolff T (2002). Importance of hemagglutinin glycosylation for the biological functions of influenza virus. Virus Res.

[12] Vallbracht M, Rehwaldt S, Klupp BG, Mettenleiter TC, Fuchs W (2018). Functional role of N-linked glycosylation in Pseudorabies virus glycoprotein gH. J Virol.

[13] Eichler R, Lenz O, Garten W, Strecker T (2006). The role of single N-glycans in proteolytic processing and cell surface transport of the Lassa virus glycoprotein GP-C. Virology journal.

[14] Vigerust DJ, Shepherd VL (2007). Virus glycosylation: role in virulence and immune interactions. Trends Microbiol.

[15] Hanna SL, Pierson TC, Sanchez MD, Ahmed AA, Murtadha MM, Doms RW (2005). N-linked glycosylation of west Nile virus envelope proteins influences particle assembly and infectivity. J Virol.

[16] Bagdonaite I, Wandall HH (2018). Global aspects of viral glycosylation. Glycobiology.

[17] Luo S, Hu K, He S, Wang P, Zhang M, Huang X, Du T, Zheng C, Liu Y, Hu Q (2015). Contribution of N-linked glycans on HSV-2 gB to cell-cell fusion and viral entry. Virology.

[18] Wanzeck K, Boyd KL, McCullers JA (2011). Glycan shielding of the influenza virus hemagglutinin contributes to immunopathology in mice. Am J Respir Crit Care Med.

[19] Job ER, Deng YM, Barfod KK, Tate MD, Caldwell N, Reddiex S, Maurer-Stroh S, Brooks AG, Reading PC (2013). Addition of glycosylation to influenza A virus hemagglutinin modulates antibody-mediated recognition of H1N1 2009 pandemic viruses. J Immunol.

[20] Lennemann NJ, Rhein BA, Ndungo E, Chandran K, Qiu X, Maury W (2014). Comprehensive functional analysis of N-linked glycans on Ebola virus GP1. MBio.

[21] McGinnes L, Sergel T, Reitter J, Morrison T (2001). Carbohydrate modifications of the NDV fusion protein heptad repeat domains influence maturation and fusion activity. Virology.

[22] Hu A, Cathomen T, Cattaneo R, Norrby E (1995). Influence of N-linked oligosaccharide chains on the processing, cell surface expression and function of the measles virus fusion protein. J Gen Virol.

[23] von Messling V, Cattaneo R (2003). N-linked glycans with similar location in the fusion protein head modulate paramyxovirus fusion. J Virol.

[24] Moll M, Kaufmann A, Maisner A (2004). Influence of N-glycans on processing and biological activity of the Nipah virus fusion protein. J Virol.

[25] Carter JR, Pager CT, Fowler SD, Dutch RE (2005). Role of N-linked glycosylation of the Hendra virus fusion protein. J Virol.

[26] Aguilar HC, Matreyek KA, Filone CM, Hashimi ST, Levroney EL, Negrete OA, Bertolotti-Ciarlet A, Choi DY, McHardy I, Fulcher JA, Su SV, Wolf MC, Kohatsu L, Baum LG, Lee B (2006). N-glycans on Nipah virus fusion protein protect against neutralization but reduce membrane fusion and viral entry. J Virol.

[27] Niwa H, Yamamura K, Miyazaki J (1991). Efficient selection for high-expression transfectants with a novel eukaryotic vector. Gene.

[28] Schneider CA, Rasband WS, Eliceiri KW (2012). NIH Image to ImageJ: 25 years of image analysis. Nat Methods.

[29] Fischer K, Groschup MH, Diederich S (2020). Importance of endocytosis for the biological activity of Cedar virus fusion protein. Cells.

[30] Watt A, Moukambi F, Banadyga L, Groseth A, Callison J, Herwig A, Ebihara H, Feldmann H, Hoenen T (2014). A novel life cycle modeling system for Ebola virus shows a genome length-dependent role of VP24 in virus infectivity. J Virol.

[31] Meulendyke KA, Wurth MA, McCann RO, Dutch RE (2005). Endocytosis plays a critical role in proteolytic processing of the Hendra virus fusion protein. J Virol.

[32] Diederich S, Moll M, Klenk HD, Maisner A (2005). The Nipah virus fusion protein is cleaved within the endosomal compartment. J Biol Chem.

[33] Bradel-Tretheway BG, Liu Q, Stone JA, McInally S, Aguilar HC (2015). Novel functions of Hendra virus G N-glycans and comparisons to Nipah virus. J Virol.

[34] Biering SB, Huang A, Vu AT, Robinson LR, Bradel-Tretheway B, Choi E, Lee B, Aguilar HC (2012). N-Glycans on the Nipah virus attachment glycoprotein modulate fusion and viral entry as they protect against antibody neutralization. J Virol.

[35] Segawa H, Yamashita T, Kawakita M, Taira H (2000). Functional analysis of the individual oligosaccharide chains of Sendai virus fusion protein. J Biochem.

[36] Zimmer G, Trotz I, Herrler G (2001). N-glycans of F protein differentially affect fusion activity of human respiratory syncytial virus. J Virol.

[37] Sagar M, Wu X, Lee S, Overbaugh J (2006). Human immunodeficiency virus type 1 V1–V2 envelope loop sequences expand and add glycosylation sites over the course of infection, and these modifications affect antibody neutralization sensitivity. J Virol.

[38] Lavie M, Hanoulle X, Dubuisson J (2018). Glycan shielding and modulation of hepatitis C virus neutralizing antibodies. Front Immunol.

[39] Sommerstein R, Flatz L, Remy MM, Malinge P, Magistrelli G, Fischer N, Sahin M, Bergthaler A, Igonet S, Ter Meulen J, Rigo D, Meda P, Rabah N, Coutard B, Bowden TA, Lambert PH, Siegrist CA, Pinschewer DD (2015). Arenavirus glycan shield promotes neutralizing antibody evasion and protracted infection. PLoS Pathog.

[40] Abe Y, Takashita E, Sugawara K, Matsuzaki Y, Muraki Y, Hongo S (2004). Effect of the addition of oligosaccharides on the biological activities and antigenicity of influenza A/H3N2 virus hemagglutinin. J Virol.

