# Supplementary material for: The role of N-linked glycosylation in proteolytic processing and cell surface transport of the Cedar virus fusion protein
Source: Virol J. 2022 Aug 23;19:136. doi: 10.1186/s12985-022-01864-5 (PMC9400332; doi:10.1186/s12985-022-01864-5)
Supplement: Supplementary file 1 — Additional file 1: Sequence information for codon-optimized CedV F HAtag gene. Start and stop codon are highlighted in bold and are underlined. CedV F gene was codon-optimized according to the human codon usage bias and synthesized by Gene Art. Additionally, the coding information for an HA-Tag was included. The CedV F gene was then subcloned into the pCAGGS expression vector using the restriction enzymes SacI and NheI (indicated in italics and underlined). [file 12985_2022_1864_MOESM1_ESM.pdf]

Additional file 1

Sequence information for codon-optimized CedV F HAtag gene

CedV F HA tag

TTC*GAGCTCT*AAAGTAATCTCAACGCCACC**ATG**AGCAACAAGCGGACCACCGTGCTGATCAT
CATCAGCTACACCCTGTTCTACCTGAACAACGCCGCCATCGTGGGCTTCGACTTCGACAAGC
TGAACAAGATCGGCGTGGTGCAGGGCAGAGTGCTGAACTACAAGATCAAGGGCGACCCCAT
GACCAAGGACCTGGTGCTGAAGTTCATCCCCAACATCGTGAACATCACCGAGTGCGTGCGG
GAACCCCTGAGCAGATACAATGAGACAGTGCGGAGACTGCTGCTGCCCATCCACAATATGC
TGGGCCTGTATCTGAACAACACCAACGCCAAGATGACCGGCCTGATGATCGCCGGCGTTAT
CATGGGAGGAATCGCCATCGGCATTGCCACCGCCGCTCAGATCACAGCCGGATTTGCCCTG
TACGAGGCCAAGAAGAACACCGAGAACATCCAGAAGCTGACCGACAGCATCATGAAGACCC
AGGACAGCATCGACAAACTGACCGACTCTGTGGGCACCAGCATCCTGATCCTGAACAAGCT
GCAGACCTACATCAACAATCAGCTGGTGCCTAACCTGGAACTGCTGAGCTGCAGGCAGAAC
AAGATTGAGTTCGACCTGATGCTGACCAAGTATCTGGTGGACCTGATGACCGTGATCGGCC
CCAATATCAACAACCCCGTGAACAAGGACATGACCATCCAGAGCCTGAGCCTGCTGTTCGAC
GGCAACTACGATATCATGATGAGCGAGCTGGGCTATACCCCTCAGGACTTCCTGGATCTGA
TCGAGAGCAAGAGCATCACCGGCCAGATCATCTACGTGGACATGGAAAACCTGTACGTGGT
CATCCGGACCTACCTGCCTACACTGATCGAGGTGCCCGACGCACAGATCTACGAGTTCAACA
AGATCACCATGTCCAGCAACGGCGGCGAGTACCTGAGCACAATCCCCAACTTTATCCTGATT
CGGGGCAACTACATGTCCAACATCGACGTGGCCACCTGTTACATGACAAAGGCCAGCGTGA
TCTGCAACCAGGACTACAGCCTGCCTATGAGCCAGAACCTGCGGAGCTGTTACCAGGGCGA
GACAGAGTACTGTCCCGTGGAAGCCGTGATCGCCAGCCACTCTCCTAGATTCGCCCTGACC
AACGGCGTGATCTTCGCCAACTGCATCAACACCATCTGCCGGTGCCAGGACAACGGCAAGA
CCATCACACAGAACATCAACCAGTTCGTGTCCATGATCGACAACAGCACCTGTAACGACGTG
ATGGTGGACAAGTTCACCATCAAAGTGGGCAAGTACATGGGCCGCAAGGACATTAACAACA
TCAATATCCAGATCGGCCCGCAGATCATCATTGACAAAGTGGACCTGTCCAATGAGATTAAC
AAGATGAACCAGAGCCTCAAGGACTCCATCTTCTACCTGCGGGAAGCCAAGCGGATCCTGG
ACTCCGTGAATATCAGCCTGATCAGCCCCAGCGTGCAGCTGTTCCTGATCATTATCTCCGTG
CTGTCCTTCATCATCCTGCTCATCATTATCGTGTACCTGTACTGCAAGAGCAAGCACAGCTA
CAAGTACAACAAGTTCATCGACGACCCCGACTACTACAACGACTACAAGAGAGAGCGGATCA
ACGGCAAAGCCAGCAAGTCCAACAACATCTACTACGTGGGCGACTACCCATACGATGTTCCA
GATTACGCT**TAA**CAATCGATAATCTAA*GCTAGC*AGAT

Start and stop codon are highlighted in bold and are underlined. CedV F gene was codon-optimized according to the human codon usage bias and synthesized by Gene Art. Additionally, the coding information for an HA-Tag was included. The CedV F gene was then subcloned into the pCAGGS expression vector using the restriction enzymes SacI and NheI (indicated in italics and underlined).
